# Early risk stratification in patients with oncological and hematological malignancies in the emergency department

**DOI:** 10.1186/cc13232

**Published:** 2014-03-17

**Authors:** A Rast, D Steiner, A Kutz, M Bargetzi, B Mueller, P Schuetz

**Affiliations:** 1 Kantonsspital Aarau, Switzerland

## Introduction

There are no well-validated risk scores for patients with oncological and hematological malignancies presenting to the emergency department (ED). Previous research found the prognostic blood biomarker pro-adrenomedullin (proADM) to be associated with infection-related complications and mortality and thus may be helpful in managing febrile patients with malignancies. Yet the prognostic value of proADM in general oncological patients presenting to the ED remains unclear. Herein, the objective of this study is to evaluate the prognostic potential of proADM and clinical parameters in a consecutive cohort of patients with oncological and hematological malignancies with regard to ICU admission and 30-day mortality.

## Methods

We enrolled all consecutive patients with oncological and hematological malignancies seeking ED care at a tertiary care hospital from February 2013 to October 2013. We prospectively collected various clinical features, and measured blood parameters including proADM upon admission. To assess outcomes, data from electronic medical records - that is, ICU admission, length of stay (LOS), and postacute care location - were used and we contacted all patients 30 days after hospital admission. Logistic regression models with area under the receiver operating curve (AUC) were used to assess association of baseline parameters and outcomes.

## Results

We included a total of 469 patients with oncological and hematological malignancies, of whom 8.9% (*n *= 42) were admitted to the ICU and 18.7% (*n *= 88) did not survive until the 30-day follow- up. There was a strong association of initial proADM levels and 30-day mortality risk (odds ratio (OR) per 10-fold increase 9.9, 95% CI 4.3, 22.9) with an AUC of 0.67 (95% CI 0.60, 0.74). This association remained significant after multivariate adjustment for initial vital signs (blood pressure, pulse, temperature) and comorbidities (chronic heart failure, chronic obstructive pulmonary disease, diabetes, coronary heart disease) with an adjusted OR of 9.0 (95% CI 3.1, 26.4). There was also a significant association of proADM and LOS (adjusted regression coefficient per 10-fold increase: 6.6, 95% CI 2.0, 11.2).

## Conclusion

This study including consecutive patients with oncological and hematological malignancies found a moderate association of proADM with 30-day mortality and LOS. proADM in combination with clinical parameters may help to improve site-of-care decisions for these patients in the future

